# *Agaricus bisporus* Polysaccharides Ameliorates Behavioural Deficits in D-Galactose-Induced Aging Mice: Mediated by Gut Microbiota

**DOI:** 10.3390/foods12020424

**Published:** 2023-01-16

**Authors:** Hui Duan, Jinwei Li, Liuping Fan

**Affiliations:** 1 State Key Laboratory of Food Science and Technology, Jiangnan University, Wuxi 214122, China; 2 School of Food Science and Technology, Jiangnan University, Wuxi 214122, China; 3 National Engineering Research Center for Functional Food, Jiangnan University, Wuxi 214122, China

**Keywords:** *Agaricus bisporus*, polysaccharides, gut microbiota, Bacteroides, SCFAs, anti-aging effect

## Abstract

White button mushroom polysaccharide (WMP) has various health-promoting functions. However, whether these functions are mediated by gut microbiota has not been well explored. Therefore, this study evaluated the anti-aging capacity of WMP and its effects on the diversity and composition of gut microbiota in D-galactose-induced aging mice. WMP significantly improved locomotor activity and the spatial and recognition memory of the aging mice. It also alleviated oxidative stress and decreased the pro-inflammatory cytokine levels in the brain. Moreover, WMP increased α-diversity, the short-chain fatty acid (SCFA) level and the abundance of beneficial genera, such as *Bacteroides* and *Parabacteroides*. Moreover, its effect on *Bacteroides* at the species level was further determined, and the enrichments of *B. acidifaciens, B. sartorii* and *B. stercorirosoris* were found. A PICRUSt analysis revealed that WMP had a greater impact on the metabolism of carbon, fatty acid and amino acid, as well as the MAPK and PPAR signaling pathway. In addition, there was a strong correlation between the behavioral improvements and changes in SCFA levels and the abundance of *Bacteroides*, *Parabacteroides, Mucispirillum* and *Desulfovibrio* and *Helicobacter*. Therefore, WMP might be suitable as a functional foods to prevent or delay aging via the directed enrichment of specific species in *Bacteroides*.

## 1. Introduction

Aging is an inevitable process characterized by a gradual decline in physical and physiological functions [1,2]. Increasing evidence supports that intestinal microbiota plays a vital role in aging processes, especially in developing cognitive decline and neurodegenerative diseases [3]. There is a close relationship between aging and the microbiota-gut-brain-axis [4,5]. For example, transplanting the gut microbiota of young mice into old mice can counteract the age-associated impairments in cognitive behavior [3]. Supplementation of a probiotic cocktail, containing three *Lactobacillus* strains and one *Bifidobacterium* strain, can improve memory and behavior in aged mice by regulating the gut microbiota and inhibiting inflammation [6]. In addition, increasing polysaccharide or fiber intake can decrease the risk of cognitive decline and neurodegenerative diseases by regulating the gut microbiota and metabolites [7]. The increased abundance of *Faecalibacterium prausnitzii, Eubacterium and Roseburia*, induced by the Mediterranean diet, were positively associated with several markers of improved cognitive function [8].

White button mushroom (WM, *Agaricus bisporus*) is one of the most common edible mushrooms in the world, representing 15% of global mushroom production [9]. It has been an important component of the human diet for over 200 years due to its delicious taste and high nutritional value [10,11,12]. The polysaccharide of white button mushrooms (WMP), an important active compound in WM, is the mixture of mannogalactan, α-D-glucan and β-D-glucans [13]. In recent years, several health-promoting functions of WMP, such as anti-aging, anti-inflammatory, and immunoregulation, have been reported. For example, WMP had potential anti-aging effects on the brain, liver, kidney and skin in the D-galactose-induced aging mice, possibly by enhancing the antioxidant status, reducing the lipid peroxidation and improving the lipid metabolism [14,15].

However, whether the anti-aging functions of WMP are mediated by gut microbiota has not been well explored. Therefore, this study evaluated the anti-aging capacity of WMP and its effects on the diversity and composition of gut microbiota in D-gal-induced aging mice. The outcomes of this study demonstrated that WMP has potential as a functional food to delay aging processes or alleviate cognitive decline.

## 2. Materials and Methods

### 2.1. Extraction of the WMP

Dried white button mushroom was purchased from a local market in Inner Mongolia, China. After grinding to the powders (60 mush), the WMP was extracted (m/w = 1:20) twice at 100 °C for 1.5 h, and precipitated with 75% ethanol at 4 °C for 18 h. The protein was removed from precipitation using the Sevag reagent. The sample was dialyzed with 3500 Da dialysis bags at 4 °C for 60 h, changing water five times. Finally, the soluble WMP was obtained by freeze-drying (Labconco, US).

### 2.2. Animal and Experimental Design

**Figure 1:**
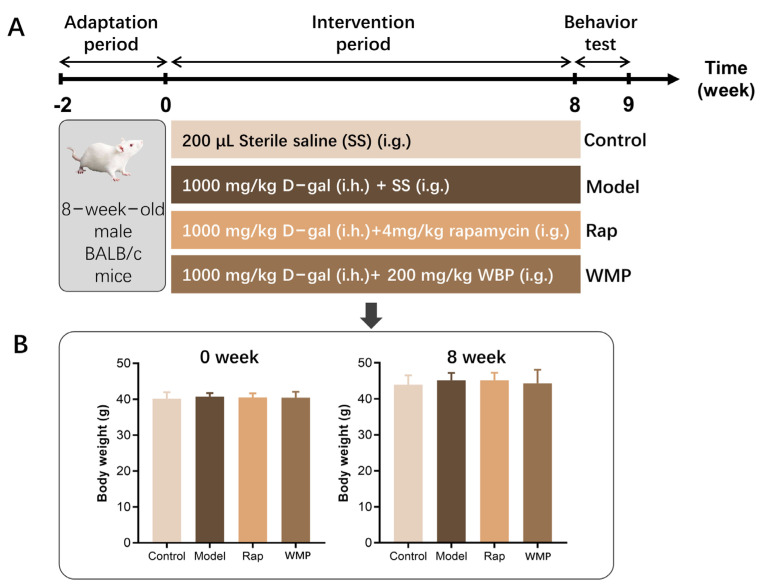
The design scheme of the animal experiment (**A**) and the effects of WMP on body weights of D-gal-induced aging mice (**B**). i.g. intragastric; i.h., hypodermic injection.

The 8-week-old male BALB/c mice were purchased from Gempharmatech Co., Ltd., (Nanjing, China), and raised in a specified pathogen free (SPF) environment with constant temperature (24 ± 1 °C) and humidity (50 ± 10%), and a 12 h light-dark cycle lighting system for the two-week adaptation period. The forty mice were then randomly divided into four groups (n = 10): control, model, WMP and rapamycin (Rap) group (Figure 1A). The total experimental period was 8 weeks. The body weight was recorded once a week. After fasting for 12 h, the mice were sacrificed and the brain and the colonic content were quickly removed and quenched in liquid nitrogen and stored at −80 °C for the following analysis.

All experimental operations are carried out following the measures for the Administration of Experimental Animals of Jiangnan University, and all animal ethics are approved by the Committee of Experimental Animal Welfare Ethics of Jiangnan University (JN.No20220530b0960905).

### 2.3. Behavioral Tests

The behavioral tests were performed using EthoVision software (Noldus, Netherlands). All of the apparatuses were cleaned with 75% alcohol after each mouse testing to avoid olfactory cues.

**Figure 2:**
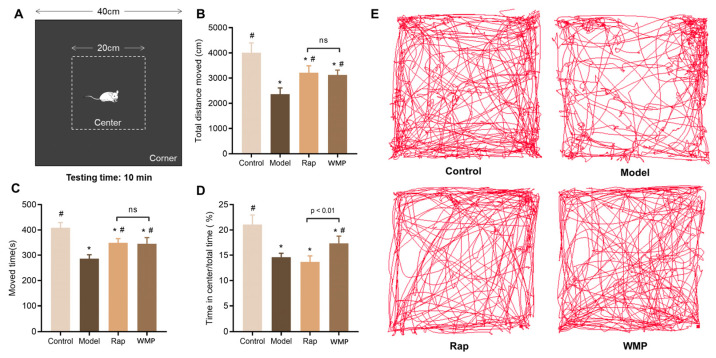
The effects of WMP on locomotor activity and anxiety-like behavior of mice. (**A**) Schematic representation of the open field test, (**B**–**D**) The total distance moved, moved times and the number of times the mice entered the center the open field, respectively, (**E**) The moving trajectory of mice in each group. * *p* < 0.05 vs control group and # *p* < 0.05 vs model group; ns: not significant.

*Open Field Test (OFT).* The OFT was performed to assess the basic locomotor activity and anxiety-like behavior [16,17]. Each mouse was allowed to move and explore freely in an open field box (black, L40 × W40 × H40 cm) for 10 min (Figure 2A). The area 20 × 20 cm in the middle is defined as the center zone, and the rest is the corner zone. A camera placed above the box was used to record its movement. Data on the total distance moved, speed, and time spent in the center zone of the field was recorded and analyzed [16,17].

**Figure 3:**
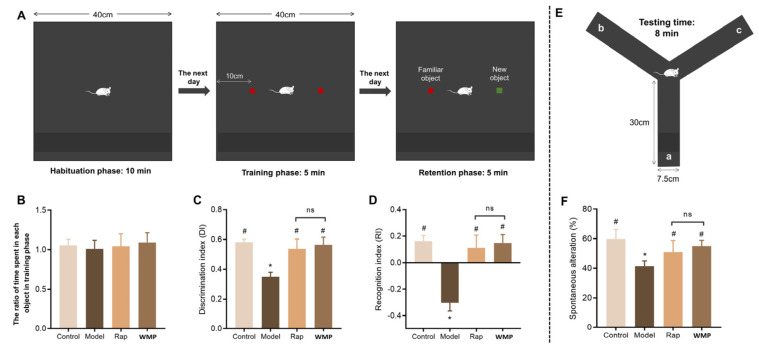
The effects of WMP on short-term spatial and recognition memory of mice. (**A**) Schematic representation of NORT, (**B**–**D**) The ratio of time spent in exploring each object in the training phase, DI and RI, respectively, (**E**) Schematic representation of the YMT, (**F**) Spontaneous alteration (%). * *p* < 0.05 vs control group and # *p* < 0.05 vs model group; ns: not significant.

*New Object Recognition Test (NORT).* The NORT was performed as described previously with minor modifications, which involved shortening the duration of the habituation phase [18]. The mice were habituated to a black box (L40 × W40 × H40 cm) for 10 min in a dim environment (Figure 3A, habituation phases). The next day they were put back into the same box with two red wood cylinders separately positioned 10 cm away from a wall. Each mouse was permitted to explore the box and the two cylinders for 5 min (training phases). The baseline performance was expressed as the ratio of time spent exploring each of the cylinders. The next day, one of the two red cylinders was replaced by a novel one, a green wood rectangular solid. The time spent exploring each object was recorded in a 5-min period (retention phases). The memory function was evaluated by the discrimination index (DI) and recognition index (RI). The equations are as follows:

DI= New object exploration time/total exploration time

RI = (new object exploring time−familiar object exploring time)/total exploring time

*Y-maze test (YMT).* The YMT is a quick and easy spatial memory test that can be reflected by the natural tendency to spontaneously choose alternate arms in the maze [19]. The YMT was conducted as previously described [20,21]. In brief, the Y-maze apparatus consists of three black plastic arms (L30 × W7.5 × H15 cm, Figure 3E). Each mouse was allowed to explore the three arms for 8 min. Entry into three different arms in succession was defined as one right consecutive alternation, including the ABC, ACB, BAC, BCA, CAB or CBA arms. The number of right alterations and total arm entries were tracked and analyzed. The spontaneous alteration ratio was calculated from the following equation: Spontaneous alteration (%) = [(number of right consecutive alterations)/(total number of arm entries—2)] × 100% [21].

### 2.4. Determination of Biochemical Markers of Oxidative Stress in the Brain

The levels of methane dicarboxylic aldehyde (MDA) and glutathione (GSH), and the activity of superoxide dismutase (SOD) in the mice brain were measured using commercially available kits (Nanjing Jiancheng Bioengineering Co. Ltd., Nanjing, China) following the manufacturer’s instructions.

### 2.5. Determination of Pro-Inflammatory Cytokine in the Brain

The levels of pro-inflammatory cytokines, including TNF-α, IL-1β and IL-6, in the brain were determined using commercially available kits (SenBeiJia Biological Technology Co., Ltd., Nanjing, China) following the manufacturer’s operating instructions.

### 2.6. 16S rRNA Sequencing Analysis of Gut Microbiota

The microbial genomic DNA was extracted using a FastDNA SPIN kit for faeces (MP Biomedicals). The V3-V4 region was amplified using the previously reported primers 341F and 806R [22]. In addition, we used species-specific primers to determine the species level relative abundance of *Bifidobacteria* (*groEL*) and *Bacteroides* (*rpsD*) referenced in previous studies [23,24]. PCR products were purified by a DNA Purification Kit (BioMIGA, San Diego, CA, USA), and sequenced using a Miseq sequencer (Illumina, Inc., San Diego, CA, USA; Illumina Miseq PE300). Data was analyzed using Qiime2 software and the MicrobiomeAnalyst online website (https://www.microbiomeanalyst.ca/MicrobiomeAnalyst/home.xhtml (accessed on 5 December 2022)). Linear discriminant analysis effect size (LEfSe) was applied to screen the potential significant differences between groups. Phylogenetic Investigation of Communities by Reconstruction of Unobserved States (PICRUSt) was used to predict the functional profiles of the gut microbiota.

### 2.7. Determination of SCFAs Level

We followed a previously published method for pretreatment of colonic content samples (50 mg) [25]. The SCFAs levels were analyzed using a GC-MS system with a flame ionization detector [25]. The GC column oven was initially maintained at 100 °C and increased to 140 °C within 5.3 min, then elevated to 200 °C within 1 min, and held for 3 min. The carrier gas was helium (He), with a flow rate of 0.89 mL/min.

### 2.8. Statistical Analysis

The experimental data of this subject are expressed as “mean ± standard deviation” (Mean ± SD), and one-way ANOVA was used to analyze the difference between groups with *p* < 0.05 indicating significant differences using Graphpad Prism 8.3 software (Boston, MA, USA).

## 3. Results

### 3.1. The Effects of WMP on the Body Weight of Mice

As shown in Figure 1B, the body weights of mice in all four groups increased by about 4 g during the 8- week experimental period, and no significant differences were observed between the groups (*p* > 0.05), indicating that D-gal and WMP, at the dose used in the study, had no significant effect on body weight.

### 3.2. The Effects of WMP on Locomotor Activity and Anxiety-Like Behavior of Mice

The OFT was normally used to assess the basic locomotor activity and anxiety-like behavior in mice [16]. D-gal would induce anxiety-like behavior as decreased time spent in the central zone. As shown in Figure 2, the D-gal induced mice to travel less distance, move slower, and spend less time in the center than the mice in the control group (*p* < 0.05). In contrast, the administration of WMP or Rap increased these three indexes to levels that were almost equal to those of mice in the control group. These results suggest that behavioural deficits in locomotor activity and anxiety-like behavior caused by D-gal could be alleviated by WMP or Rap in mice.

### 3.3. The Effects of WMP on the Short-Term Recognition Memory of Mice

In the NORT, the mice will spend more time exploring a novel object because they like to access novel things, so when the familiar object was replaced with a new one, the mice will try to explore the new object if the memory function is good [18]. In the training phase, the ratio of time spent exploring each of the cylinders in all four groups was about 1.0, with no significant difference (*p* > 0.05, Figure 3B). The discrimination index (DI) and recognition index (RI) were used to represent the recognition memory function. The DI of mice in the control group was almost twice that of the mice in the model group (*p* < 0.05), and the D-gal-treated mice had negative RI (Figure 3C,D). The mice in WMP and Rap groups showed significant increases in DI and RI compared with the model group mice (*p* < 0.05). There were no significant differences between the control, WMP and Rap groups. These results demonstrated that WMP significantly alleviated the disfunction of recognition memory induced by D-gal in mice.

### 3.4. The Effects of WMP on Short-Term Spatial Memory of Mice

In the Y-maze, spontaneous alternation refers to the natural tendency of mice to choose alternate arms spontaneously, which was used to test their spatial memory. Compared to the control group, the model group showed a significantly decreased spontaneous alternation (*p* < 0.05, Figure 3F). When the mice were orally administrated WMP or Rap, spontaneous alternation was significantly increased to 55.05% and 50.98% (*p* < 0.05), respectively, almost to the level of the control group.

### 3.5. The Effects of WMP on the Antioxidant Index

**Figure 4:**
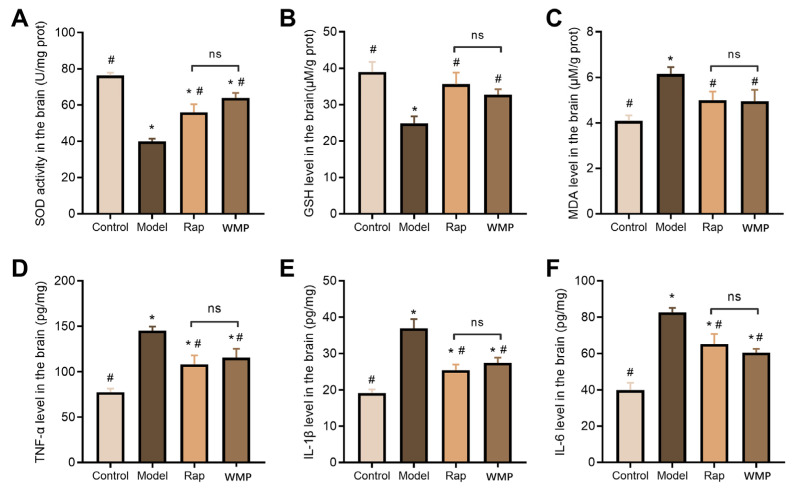
The effects of WMP on the antioxidant index and inflammatory cytokine in the brain. (**A**–**C**) SOD activity, GSH and MDA levels, respectively, (**D**–**F**) the levels of TNF-α, IL-1β and IL-6, respectively. * *p* < 0.05 vs control group and # *p* < 0.05 vs model group; ns: not significant.

The changes in the antioxidant index in the brain are presented in Figure 4A–C. Compared with the control group mice, the GSH level and SOD activity were dramatically reduced, and the MDA level increased in the brain samples of the mice in the model group (*p* < 0.05). These alterations were significantly reversed by WMP or Rap (*p* < 0.05).

### 3.6. The Effects of WMP on the Inflammatory Cytokine

As shown in Figure 4D–F, D-gal markedly increased the levels of pro-inflammatory cytokines, including TNF-a, IL-1β and IL-6 (*p* < 0.05). The increased pro-inflammatory cytokines in the model group were significantly reduced by the oral administration of WMP or Rap (*p* < 0.05).

### 3.7. The Effects of WMP on Gut Microbiota

**Figure 5:**
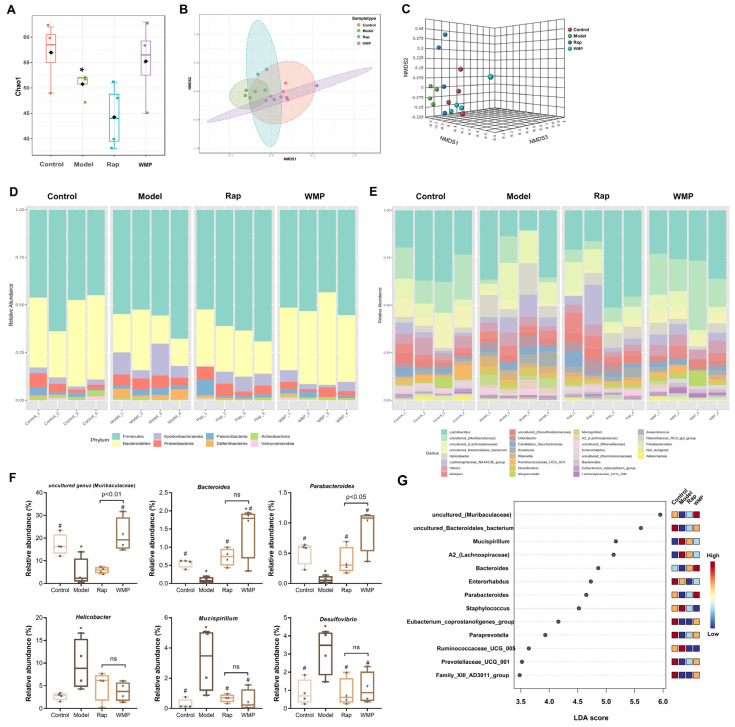
Effects of WMP on the diversity and composition of gut microbiota in mice. (**A**) Boxplots of the Chao 1 index for the gut microbiota. (**B**,**C**) The 2D and 3D plots of the scores of the principal component analyses of the gut microbiota. (**D**–**F**) The relative abundance of the gut microbiota at the phylum and genus levels. (**G**) The significantly changed genus screened by the random forest method. * *p* < 0.05 vs control group and # *p* < 0.05 vs model group; ns: not significant.

Compared with the control group, the Chao 1 index of the gut microbiota was significantly decreased in the model group, indicating that D-gal significantly reduced the α diversity (Figure 5A). The restoration of the α diversity was found in the mice in the WMP group. The β-diversity was used to compare differences between microbial community profiles. Distinct variances of β-diversity were observed (*p* = 0.006) between groups (Figure 5B,C). Specifically, the overall structure of the gut microbiota in the WMP group was different from that in the model group, but similar to that in the control group.

The four predominant bacterial phyla in the mice in the control group were Firmicutes, Bacteroidetes, Proteobacteria and Epsilonbacteraeota (Figure 5C). D-gal-induced mice had a significantly decreased abundance of Bacteroidetes, and a greater amount of Epsilonbacteraeota and Deferribacteres, whereas the administration of WMP had an opposite effect. As shown in Figure 5D–G, the relative abundance of 15 genera, such as uncultured_(Muribaculaceae), *Mucispirillum, Bacteroides, Enterorhabdus* and *Parabacteroides,* were significantly changed (*p* < 0.05). The abundances of uncultured_(Muribaculaceae), *Bacteroides* and *Parabacteroides* were reduced in the model group, while the abundance of harmful genera, including *Helicobacter, Mucispirillun,* and *Desulfovibrio,* were dramatically increased (*p* < 0.05). After WMP administration, their abundance would return to the control levels.

**Figure 6:**
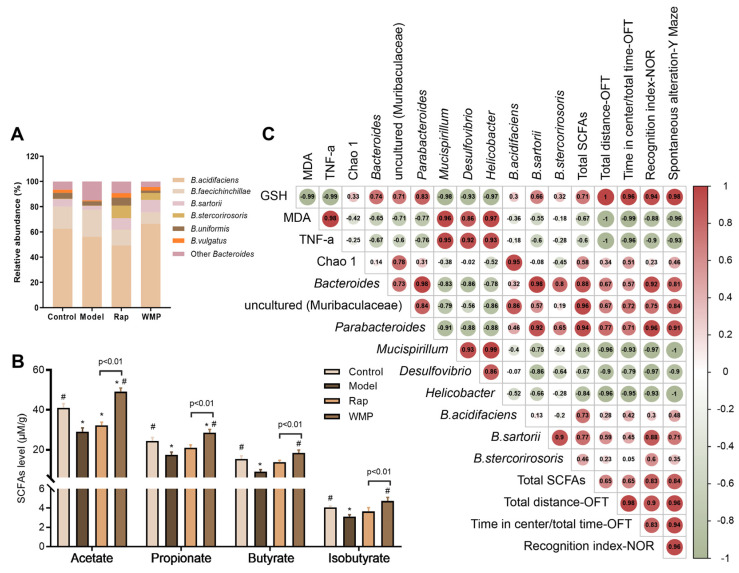
Effects of WMP on the SCFAs level and the Spearman correlation analysis. (**A**) Effects of WMP on the *Bacteroides*, (**B**) The SCFAs levels, and (**C**) the Spearman correlation analysis. The colors and values indicate the distribution of the Spearman’s correlation coefficients. * *p* < 0.05 vs control group and # *p* < 0.05 vs model group.

As the relative abundance of *Bacteroides* was high and changed dramatically, the effect of WMP on *Bacteroides* at the species level was further determined (Figure 6A). The top 5 species in the *Bacteroides* genus of mice in the control group were *Bacteroides acidifaciens (B. acidifaciens), B. faecichinchillae, B. sartorii, B. stercorirosoris* and *B.uniformis*, and the enrichments of the *B. acidifaciens, B. sartorii* and *B. stercorirosoris* were found in the WMP group.

### 3.8. The Effects of WMP on SCFA Levels in Mice Faeces

The top three SCFAs in the faecal samples were acetic acid, propionic acid, and butyric acid (Figure 6B). Compared with mice in the control group, the three SCFAs in the mice faeces in the model group were markedly reduced (*p* < 0.05). WMP treatment led to 68.96% and 64.59% increases in the levels of acetic acid and propionic acid, respectively, compared with the corresponding increases of only 11.07% and 21.01%, respectively, in the Rap group, indicating that WMP had a stronger effect on SCFAs than the Rap group.

### 3.9. Spearman Correlation Analysis

A Spearman correlation analysis revealed that the behavioral improvements were not only strongly correlated with the indexes of oxidative stress and inflammatory cytokine in the brain, but were also related to the abundances of specific gut microbial genera and SCFAs levels. Specifically, the locomotor activity and anxiety-like behavior were strongly negatively correlated with the abundances of *Mucispirillum* and *Helicobacter* (|r| > 0.90); spatial and recognition memory was positively correlated with the abundances of *Bacteroides*, *Parabacteroide* and SCFAs levels, while they were negatively correlated with the abundances of *Mucispirillum, Desulfovibrio,* and *Helicobacter* (|r| > 0.80, Figure 6C).

### 3.10. Effects of WMP on the Functional Profile of the Gut Microbiota

**Figure 7:**
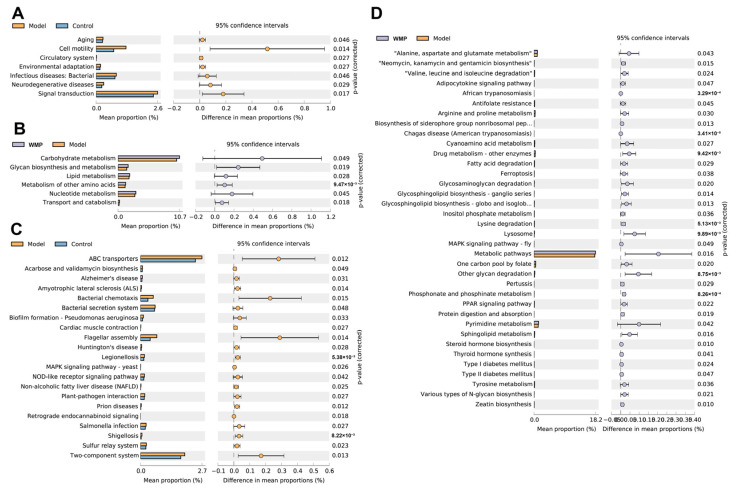
Effects of WMP on the functional profile of the gut microbiota. (**A**,**B**) the enriched KEGG pathways at level 2, (**C**,**D**) the enriched KEGG pathways at level 3.

The results of PICRUSt-predicted functional profiles showed that D-gal markedly enriched 7 and 21 KEGG pathways at level 2 and level 3, respectively, including aging, cell motility, neurodegenerative diseases (Alzheimer’s and Huntington’s diseases) and the MAPK signaling pathway (Figure 7). WMP markedly affected 6 and 36 KEGG pathways at level 2 and level 3, respectively, including the metabolism of carbohydrate, lipid, amino acids (alanine, aspartate, glutamate, arginine and proline), MAPK, and the PPAR signaling pathway.

## 4. Discussion

Edible mushrooms have been used extensively used for thousands of years because of their nutritional and medicinal value [26,27]. Mounting evidence indicates that edible mushroom polysaccharides can regulate gut microbiota and produce functional metabolites, such as SCFAs, thereby exerting various beneficial effects [28]. Additionally, as the number of elderly people living with cognitive decline is rising, causing serious burdens for individuals and society, new strategies are required to improve quality of life [29]. It has been reported that D-gal can induce brain aging not only by causing mitochondrial dysfunction, but also by increasing oxidative stress, inflammation, and apoptosis, and finally by causing cognitive decline [30]. Therefore, the D-gal-induced brain aging model is widely used to study the anti-aging therapeutic interventions [31]. Recently, the microbiota-gut-brain axis has been reported to be involved in the progression of age-related cognitive impairment and the corresponding alterations of brain structure [32].

The causal effects of the gut microbiota on the brain and behavior have also been demonstrated in recent decades. For example, the oral administration of the probiotic *Lactobacillus rhamnosus* JB-1 alleviated anxiety- and depression-like behavior [33]. A high relative abundance of *Bacteroides* is correlated with better cognitive performance [29], increased grey matter in the brain [34], and was negatively associated with depression [35]. *Parabacteroides* can ameliorate obesity and metabolic dysfunctions via the production of succinate and secondary bile acids [36]. Its beneficial effects on depression- and anxiety-like behavioral changes were also observed, and the mechanism may be to affect the kynurenine pathway in gut-brain interaction [37]. Moreover, *Parabacteroides,* taxa most highly enriched by the ketogenic diet [38], was also associated with an increased ketones level [39]. Ketones can alter the metabolism of amino acids and neurotransmitters, such as γ-amino butyric acid (GABA), improve mitochondrial function, decrease oxidative stress, and activate the peroxisome proliferator-activated receptor (PPAR) and AMPK pathways [40,41,42]. Interestingly, *Bacteroides* and *Parabacteroides* produced large quantities of GABA via their GABA-producing pathways [35]. A 40% reduction in GABA levels in the rat prefrontal cortex was associated with reduced levels of *Bacteroides* and depressive-like behavior [43].

The relative abundance of *Bacteroides* was high and dramatically elevated by WMP, therefore, the effect of WMP on *Bacteroides* at the species level was further explored. It increased the relative abundance of *B. acidifaciens, B. sartorii* and *B. stercorirosoris*. The abundance of *B. acidifaciens* can be enriched by a high soluble fiber diet [44,45]. This species also has various beneficial functions, such as preventing obesity [46], increasing insulin sensitivity [47], and promoting the production of acetate to protect against nonalcoholic steatohepatitis development [48]. In addition, *B. acidifaciens*-monocolonized mice increase secretory IgA, generate B-cell memory, and thereby strengthen ocular mucosal barrier function [49]. Wang et al. compared the protective effects of four *Bacteroides* (*B. acidifaciens*, *B. thetaiotaomicron*, *B. dorei* and *B. uniformis*) on the liver [50]. Only *B. acidifaciens* played a protective role against liver injury via the reduction of CD95/CD95L signaling. *B. stercorirosoris* was also enriched by fungi polysaccharides from Fuzhuan Brick Tea, which can ameliorate DSS-induced ulcerative colitis in mice [51]. Furthermore, Wei et al. explored the reason for the enrichment of *B. acidifaciens* and *B. sartorii* used seaweed polysaccharides, and the result was attributed to their fucoside degradation potential [46].

The consumption of WMP can not only be associated to an increment of the abundance of beneficial bacteria, but also reduce the abundance of harmful species, such as *Mucispirillum sp.*, *Desulfovibrio sp.,* and *Helicobacter sp. Mucispirillum sp.* resides in the intestinal mucus layer of rodents and harbors some virulence traits, including a type VI secretion system and putative effector proteins [52]. The increase in the relative abundance of *Mucispirillum sp.* and *Desulfovibrio sp.*, induced by a high-fat/high-cholesterol diet, is associated with fatty liver and even liver cancer [53]. The ‘inflammatory’ type microbiota was characterized by a higher abundance of *Desulfovibrio sp.* and *Mucispirillum sp.*, which are associated with a state of intestinal inflammation and brain disorders [54]. For example, *Mucispirillum sp.* accumulation triggered Crohn’s disease and colitis in mice [55]. Meanwhile, *Mucispirillum sp.* was enriched in the patients with Parkinson’s disease [56], and it was negatively correlated with cognitive ability and positively correlated with the level of IL-6 in the brain cortex [57]. A higher abundance of *Rikenella sp.* would induce transmissible colitis and colorectal cancer [58]. A higher abundance in *Helicobacter sp.* also presented positive correlations with hepatic injury, lipid metabolism, and fibrosis via the ileum FXR-FGF15-FGFR4 pathway [59]. *Helicobacter sp.* is associated with motor disorders in mice with Parkinson’s disease via activating asparagine endopeptidase [59].

## 5. Conclusions

In conclusion, this study demonstrates that WMP can protect against D-gal-induced behavioural deficits, especially in locomotor activity, and spatial and recognition memory in mice. These behavioral improvements were positively correlated with the abundances of *Bacteroides sp.*, *Parabacteroides sp,* and SCFAs levels, and were negatively correlated with the abundances of *Mucispirillum sp., Desulfovibrio sp.,* and *Helicobacter sp.* It is worth mentioning that WMP can enrich *B. acidifaciens*, *B. sartorii*, and *B. stercorirosoris* in mice. These results show that WMP has the potential to be used as a dietary supplement to delay aging processes and prevent age-related diseases via the directed enrichment of specific species in *Bacteroides sp*.

## Data Availability

The data presented in this study are openly available in FigShare at https://doi.org/10.6084/m9.figshare.21899439.
